# Implementation and assessment of a novel non-clinical skills curriculum for urology residents

**DOI:** 10.3389/fruro.2023.1167966

**Published:** 2023-06-13

**Authors:** Tyler Sheetz, Dinah Diab, Alicia Scimeca, Fara Bellows, David S. Sharp, Cheryl T. Lee, Tasha Posid

**Affiliations:** Department of Urology, The Ohio State University Wexner Medical Center, Columbus, OH, United States

**Keywords:** medical education, hidden curriculum, graduate medical education (GME), urology, non-clinical skills

## Abstract

**Background:**

Urology is an increasingly competitive specialty that procures a highly selected and clinically excellent cohort of residents. However, other training needs such as leadership and professional development go underrecognized despite an identified need for formal training in these areas. The aim of this study was to implement, evaluate, and pilot a non-clinical skills curriculum, a novel individualized professional development workshop series, at a single institution.

**Methods:**

Eighteen urology residents (15/year, 3 graduates/year) participated in this study over the course of two academic years. A pre-curriculum needs assessment was completed by 15 residents in Year 1 for purposes of curriculum design. The curriculum itself was a series of 1-hour monthly workshops given by an expert speaker on topics relevant to healthcare delivery, leadership and career promotion across various contexts. Survey-based assessments tracked gains in subject knowledge and satisfaction via a pre-post test design.

**Results:**

The pre-curriculum needs assessment indicated that trainees desired additional instruction in non-clinical skills (ps>0.1) and endorsed formal teaching to ensure success in their future careers (p<0.001). Trainees reported pre- to post-curriculum gains across each individual learning topic (Mean=20%, p<0.001) with an aggregate increase in subject knowledge of 17% for senior residents and 21% for junior residents (p<0.001).

**Conclusion:**

A non-clinical skills curriculum implemented as a pilot ‘Hidden Curriculum’ for urology trainees was feasible and resulted in significant gains in non-clinical subject knowledge. Workshops were highly rated and trainees reported high satisfaction with the curriculum.

## Introduction

With a 2022 Match rate of 66% (1), urology remains one of the most competitive residency programs and thus attracts some of the most qualified medical school applicants (2). Urology residents tend to excel clinically, with a 92% residency completion rate (3) and 97% pass rate in the qualifying examination over the past five years (4). While urology residency programs are frequently evaluated on clinical (5, 6), surgical (7, 8), and research-based training (9, 10), little work has been published addressing non-clinical skills (NCS), concepts not formally taught in a residency setting but deemed useful for the career of a developing urologist, despite an identified need for training in leadership (11, 12) and career development (13) in surgical specialties.

Given that participation of urology residents in patient care impacts patient satisfaction and outcomes (14) and residents with better leadership and directional skills are more efficient in the operating room (15), we must also arm our surgical trainees with solid NCS in communication, leadership, and administration. NCS in surgery have been correlated with higher patient satisfaction scores, lower rates of burnout, greater academic success, improved patient-physician relationships (16), lower rates of adverse iatrogenic events in surgery (17), and advanced technical skills (15). Furthermore, recent reports have called for formalized leadership and NCS training in surgical residency programs (11, 12, 18, 19). In one study, a majority of practicing urologists rated NCS training as “useful” but felt that residency training left them inadequately prepared for these aspects of practice (20). A more recent review identified deficiencies in NCS training in modern urological curricula and strategies to address them (21).

Thus, this study seeks to address a critical gap in trainees’ professional development by designing, implementing, and evaluating a NCS curriculum intended to hone skills in medical leadership and strengthen related competencies as a pilot model for a feasible ‘Hidden Curriculum’ for Urology residents.

## Materials and methods

### Study sample

Over the course of two academic years, 18 urology residents participated in this prospective curriculum at a large Midwestern public academic university. In Year 1, all 15 departmental urology residents completed a pre-curriculum needs assessment, participated in planned 1-hour learning workshops (where they completed a post-timepoint evaluation), and completed an identical post-curriculum assessment. Given the success of the curriculum pilot in Year 1, it was continued in Year 2. A pre/post-curriculum assessment was not given in Year 2, since this had already been collected in Year 1 and was primarily used for curriculum design. During Year 2, again all 15 departmental residents were enrolled in the sessions; 3 post graduate year (PGY) 5 residents completed training at the end of Year 1 and 3 new PGY-1 residents entered the program in Year 2. Junior residents were defined as PGY 2-3 while senior residents were defined as PGY 4-5. This study was considered exempt by the Institutional Review Board at our institution.

### Curriculum design

In order to identify deficiencies in current training as well as to tailor the curriculum to our group of residents, a focused pre-curriculum needs assessment was completed by all 15 urology residents in our program at the start of the 2019-2020 academic year (**Table 1**). A copy of the pre-curriculum needs assessment can be found in **Supplementary File 1**.

**Table 1:** Pre-Curriculum Needs Assessment Results Urology residents (n=15) rated their current level of satisfaction/preparedness in various aspects of NCS (A) as well as their knowledge at baseline across relevant non-clinical topics (B) on a Likert scale ranging from 1-5.

(A) Survey Part 1 (n=15 residents)				
Survey Question	Juniors	Seniors	All Residents	p-value*
Current NCS level of satisfaction	3.25 (0.71)	3.50 (0.55)	3.36 (0.63)	0.002
Additional need for NCS training in residency	3.38 (1.19)	3.50 (0.55)	3.43 (0.94)	0.04
Level of formal NCS training currently provided	2.25 (0.71)	2.17 (0.75)	2.21 (0.70)	<0.001
Level of informal NCS training currently provided	3.25 (0.71)	2.83 (0.75)	3.07 (0.73)	<0.001
Additional NCS training needed to succeed as physician?	3.63 (0.52)	3.67 (0.52)	3.64 (0.50)	0.019
Non-clinical patient management currently taught?	3.00 (0.86)	2.83 (0.41)	2.93 (0.62)	<0.001
Current preparedness for running a clinic?	3.00 (0.76)	2.67 (1.21)	2.86 (0.95)	0.001
Current preparedness for large patient volumes?	3.43 (0.98)	3.50 (0.84)	3.46 (0.88)	0.047
Current preparedness for business aspects of medicine?	2.00 (1.00)	2.17 (0.75)	2.08 (0.86)	<0.001
Current preparedness to utilize other healthcare resources?	2.86 (0.90)	2.67 (1.03)	2.77 (0.93)	<0.001
Overall satisfaction with NCS training in residency	2.86 (0.69)	2.67 (0.52)	2.77 (0.60)	<0.001
Junior residents should have required NCS training	4.00 (1.07)	4.00 (1.26)	4.00 (1.11)	0.999
Senior residents should have required NCS training	4.38 (0.74)	4.17 (1.17)	4.29 (0.91)	0.263
(B) Survey Part 2 (n=15 residents)				
Survey Question	Juniors	Seniors	All Residents	p-value*
Effective Documentation	3.13 (0.52)	3.67 (0.64)	3.36 (0.63)	0.002
Advanced Directives/Delivering Bad News	2.75 (0.71)	3.33 (0.82)	3.00 (0.78)	<0.001
Teaching Skills/Feedback	2.75 (1.16)	3.67 (0.82)	3.14 (1.10)	0.012
Wellbeing/Wellness/Maintaining Passion	2.75 (1.04)	3.67 (0.82	3.08 (0.95)	0.004
MOC, CME, Professional Responsibilities	1.63 (0.74)	3.17 (0.75)	2.29 (1.07)	<0.001
Leadership Styles	3.13 (0.83)	3.80 (0.45)	3.38 (0.77)	0.014
Common Patient Complaints/Strategies	2.75 (0.71)	3.67 (0.52)	3.14 (0.77)	0.001
Job Search/Next Steps/Fellowships	2.00 (1.20)	3.67 (0.82)	2.71 (1.33)	0.003
Cultural Competency	3.38 (0.52)	3.67 (0.52)	3.50 (0.52)	0.003
Bioethics	3.50 (1.05)	3.50 (1.07)	3.50 (1.02)	0.089
Informed Consent	3.88 (0.83)	4.00 (0.00	3.98 (0.62)	0.671
(Urology) Billing and Coding	2.00 (0.76)	3.17 (0.75)	2.50 (0.94)	<0.001
Gender Issues in Medicine Surgery	3.00 (0.53)	3.17 (0.75)	3.07 (0.62)	<0.001
Urology Administration Governing Boards	1.88 (0.83)	3.17 (0.41)	2.43 (0.94)	<0.001

Table displays mean Likert rating (SD). Topics in **(B)** were utilized to design Years 1 and 2 of our curriculum.

Values represent mean Likert rating (SD) of knowledge on a scale of 1-5: 1 (novice or none) to 5 (expert).

CME, continuing medical education; MOC, maintenance of certification; NCS, non-clinical skills.

*All residents vs. “high knowledge” (or 4/5 on Likert scale).

In this assessment, residents were asked to rate their knowledge of specific topics proposed for learning sessions. This needs assessment was created internally by our department’s Education Specialist (PhD) with >11 years of expertise in survey creation and design. Evaluations also underwent iterative review by our Education Leaderhip (FB, DS, or CL), who also serve as members of our departmental Education Working Group, a 10-12 person internal committee of medical and surgical educators who review all proposed and ongoing curricula in the department on a quarterly basis. The Education Working Group and these faculty educators came up with the learning topics presented in our pre-curriculum needs assessment. Our goal was to create learning sessions for any topic not rated at a ‘high’ knowledge level, defined as 4 out of 5 on a 1-5 Likert-scale. Any topic with a mean value statistically significantly lower than 4 was created as a learning session in Year 1 or 2 (**Table 1B**).

The curriculum itself was initiated in August 2019 and consisted of a series of 1-hour monthly workshops given by an expert speaker (**Table 2**). Attendance by residents for each learning session was mandatory, barring patient care emergencies or pre-established vacation time. In Year 1, the last 2 sessions took place virtually via Zoom, given institutional restrictions on in-person gatherings due to the COVID-19 pandemic. Following each learning session, trainees filled out a short (<5 minute) timepoint assessment of the session (**Supplementary File 1**). At the end of Year 1 (2019–2020), a post-curriculum assessment identical to the pre-curriculum needs assessment, was administered electronically via REDCap (Research Electronic Data Capture) to all urology residents.

**Table 2:** Post-session timepoint assessment results for residents.

NCS Topic	Improved education/ prof. dev.	Improved teamwork abilities	Would recommend to a peer	Was intellectually challenging	Increased knowledge of subject	Allowed practicing NCS	Increased problem-solving	Improved leadership skills	Session Mean
Effective Documentation	4.35 (1.07)	3.67(1.52)	3.60(1.51)	3.40(1.34)	3.60 (1.52)	4.20(0.84)	3.80(0.84)	3.60 (1.14)	3.89(0.29)
Teaching Skills/ Feedback	4.80 (0.50)	4.60(0.70)	4.80 (0.63)	4.40 (0.97)	4.80(0.42)	3.90(1.45)	4.20(1.14)	4.60 (0.70)	4.57(0.34)
EAP/Wellness Resources	4.30(0.94)	2.75(1.71)	3..00(1.22)	3.00(1.22)	3.40(1.51)	3.60(0.89)	3.00 (1.58)	3.00(1.58)	3.11 (0.29)
Leadership Styles	3.88(0.99)	4.88(0.35)	4.88(0.35)	4.75 (0.46)	4.63(0.52)	4.25(1.38)	4.50 (0.76)	4.63(0.52)	4.47(0.36)
Job Search/Next Steps/Fellowships	4.30(0.94)	4.00 (1.22)	4.67(0.50)	4.00(1.22)	4.78(0.44)	4.11(1.17)	4.11(1.17)	4.22(1.09)	4.27(0.32)
(Urology) Billing and Coding	3.40(1.58)	3.60(1.51)	4.80 (0.42)	4.60 (0.52)	4.90(0.32)	3.90(1.29)	3.90(1.45)	3.60(1.71)	4.09 (0.59)
Gender Issues in Medicine/Surgery	4.33(1.03)	4.50 (0.84)	5.00 (0.00)	4.67(0.51)	5.00 (0.00)	4.17(0.98)	4.50(0.84)	4.67(0.52)	4.57 (0.41)
Urology Admin Governing Boards	4.35(0.99)	4.33 (0.98)	4.58(0.90)	4.58(0.67)	4.54(0.82)	4.00(1.41)	3.83(1.46)	4.54 (0.69)	4.35 (0.27)
COVID-19 Updates	4.00 (0.82)	4.00 (0.82)	4.50 (0.58)	4.25 (0.96)	4.50(0.58)	3.75(0.96)	4.00 (0.82)	4.25(0.96)	4.16(0.27)
Professionalism in Social Media	4.14(0.69)	4.00 (0.82)	4.57(0.53)	3.86 (0.69)	4.71(0.49)	3.29(1.25)	3.43(0.98)	3.57(0.79)	3.97 (0.23)
Student Loan Management	4.50(0.84)	3.33(1.63)	4.50(0.55)	4.00(1.10)	4.67(0.52)	3.50(1.52)	3.50(1.52)	3.33(1.63)	3.92 (0.57)
History of Pediatric Urology	3.83(1.17)	3.50(1.05)	3.67(1.21)	3.67(0.82)	4.50(0.55)	3.17(1.17)	3.83(1.47)	3.17(1.47)	3.54(0.51)
Private/Community Practice Panel	4.86(0.38)	4.00 (1.00)	5.00 (0.00)	4.43 (0.79)	4.86(0.38)	4.00 (1.29)	4.43(0.53)	4.57(0.53)	4.52(0.38)
**Aggregate Mean**	4.86(0.38)	4.00 (1.00)	5.00(0.00)	4.43 (0.78)	4.86(0.38)	4.00 (1.29)	4.42(0.53)	4.57(0.53)	4.55 (0.39)

**Figure 1:**
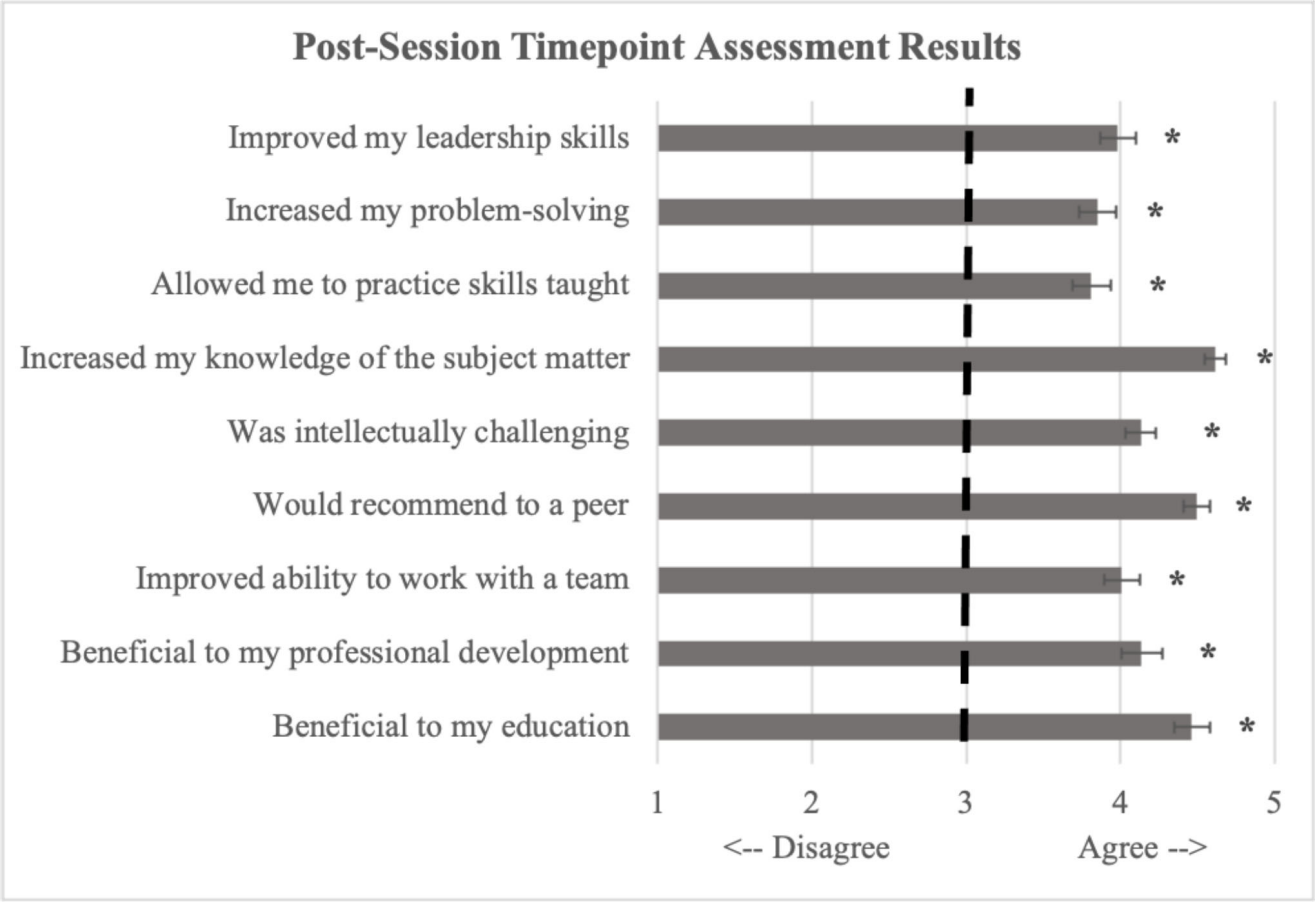
Aggregates from each column of **Table 1** were calculated and reported above. Values represent mean Likert rating (SD) responses on a scale of 1-5: 1 (disagree) to 5 (agree) with 3 representing “neutral”. As seen in the figure, the curriculum was overall rated very highly with trainees reporting that it increased their knowledge of the subject matter and that the sessions were beneficial to their education. *p<0.001.

Results from post-session assessments (administered after each learning session) stratified by topic. Following each learning session, trainees were asked to rate each statement above for that session’s topic on a Likert scale. Rightmost column represents mean Likert rating per topic for all competencies combined. See **Figure 1** for aggregates of each column.

Values represent mean Likert rating (SD) of knowledge on a scale of 1-5: 1 (novice) to 5 (expert).

EAP, employee assistance programming; NCS, non-clinical skills.

In Year 2 (2020-2021), an identical format was followed for the curriculum, with notable exceptions of all sessions taking place virtually via Zoom and no pre- or post-assessment administered (timepoint assessments only).

### Learning sessions

Learning session topics came directly from resident feedback via the pre-curriculum needs assessment. Expert speaker identification and scheduling was performed by the department’s Education Specialist (PhD) with input from Education Leadership (FB, DS, or CL). Expert speakers were all internal to our College of Medicine and were identified by the Education Leadership as knowledgeable in that topic area. The format of each session was determined by the presenter and broadly categorized into (a) primarily lecture-based or (b) “interactive” (with time for learners to engage with institutional experts and leaders), with adjunct question-and-answer sessions for both sessions.

For example, the “Urology Administration and Governing Boards” session was a Powerpoint-based lecture delivered by the program director overviewing academic and professional hierarchies and memberships with time for questions at the end. On the other hand, the “Leadership Styles” session delivered by our institution’s chief medical officer was a loosely-structured interactive discussion between the trainees and the presenter. Other sessions such as “Teaching Skills/Feedback” had elements of lecture and discussion, but was ultimately categorized as “interactive” due to >50% time being spent in a discussion format.

### Measures

Study measures consisted of surveys administered to subjects prior to participation in the curriculum in Year 1 (“pre-curriculum needs assessment”), following each learning session in Years 1 and 2, (“timepoint assessments”), and at the conclusion of the curriculum in Year 1 (“post-curriculum assessment”). We also report measures of study feasibility including cost and attendance.

### Statistical analysis

Study data were collected and managed using REDCap electronic data capture tools hosted at our institution (22). REDCap was used for survey creation, distribution, and data storage.

All data analysis was performed using SPSS Statistics software (IBM SPSS Statistics for Windows, version 27.0. Armonk, NY: IBM Corp). Data are presented as means (standard deviations) or proportions (percentages). Per study objectives, analyses were performed comparing group differences (e.g. across timepoints; or junior vs. senior residents) via chi-square tests for categorical variables, paired t-tests for continuous variables (e.g. pre- vs. post-test), or independent t-tests for continuous variables (e.g., junior vs. senior residents). Single sample t-tests were used to compare single group means (e.g., gains = post – pre) to a predetermined standard (e.g. zero). All figure error bars indicate standard error of the mean.

## Results

### Demographics

Demographics information is listed in **Table 3**. Participants were 86% Caucasian, 60% married or living with a partner, and the mean age was 28.9 years (SD=2.73). Residents were 53% male and 47% female.

**Table 3:** Demographics of Study Sample.

Demographic	Number (Percent)
Gender	
Male	8 (53%)
Female	7 (47%)
Non-Binary	–
Marital Status	
Married or living with a partner	9 (60%)
Single	6 (40%)
Race/Ethnicicty	
Caucasian	13 (87%)
Asian or South Asian	2 (13%)
Black or African American	–
Hispanic or Latino	–
Middle Eastern	–
PGY Level	
PGY-1	3 (20%)
PGY-2	3 (20%)
PGY-3	3 (20%)
PGY-4	3 (20%)
PGY-5	3 (20%)
**Mean Age in Years (SD)**	28.9 (2.73)

### Pre-curriculum needs assessment

Urology residents (n=15) were asked to rate their satisfaction/dissatisfaction with their NCS training prior to participation in the NCS Curriculum and speculate on additional training needed to prepare them for career success (**Tables 1A, B**). Prior to participating in our new curriculum, residents reported that they were only “neutral” to “slightly satisfied” with their NCS and abilities [Mean (M)=3.36/5, p=0.055)], despite the fact that they thought a “moderate amount” to “significant amount” of non-clinical training was necessary to succeed in their current position and program (M=3.43/5), as well as in their future careers (M=3.64/5, ps<0.001). Residents rated their pre-curriculum NCS training on average between “slightly unsatisfied” and “neutral” (M=2.21/5, p=0.001), with slightly more education provided informally, although residents indicated this was still a “neutral” amount (M=3.07/5, p=0.720). Junior and senior residents did not differ in their opinions (ps>0.1), except that junior residents felt they received more informal training (M_Senior_=2.83/5 vs. M_Junior_=3.25/5, p<0.001, **Table 1A**).

With respect to individual topics, residents self-reported mostly “below average” or “average” knowledge (i.e., a mean statistically less than 4 out of 5 on a 5-point Likert scale) overall (M=2.98/3, p=0.902), as well as by topic (**Table 1B**).

The trainee cohort felt the NCS Curriculum would be most beneficial for junior residents (86.7%) and senior residents (80.0%), followed by chief residents (73.3%), interns (60.0%), fellows (46.7%), and medical students (33.3%). Trainees on average “agreed” that both junior (M=4.0/5, p<0.01) and senior (M=4.28/5, p<0.01) residents should be required to participate in the curriculum (**Table 1A**).

### Learning assessments

Learning sessions spanned two academic years and both in-person and virtual formats (due to COVID-19 restrictions). We observed no difference in satisfaction with the curriculum or self-reported gains in content knowledge between the first and second academic year (ps>0.1), or between an in-person vs. virtual format (ps>0.1). Thus, learning sessions have been analyzed and reported together. Broadly, the curriculum was rated very highly in terms of key measures (**Figure 1**). Evaluation by individual learning session appear in **Table 2**.

**Figure 2:**
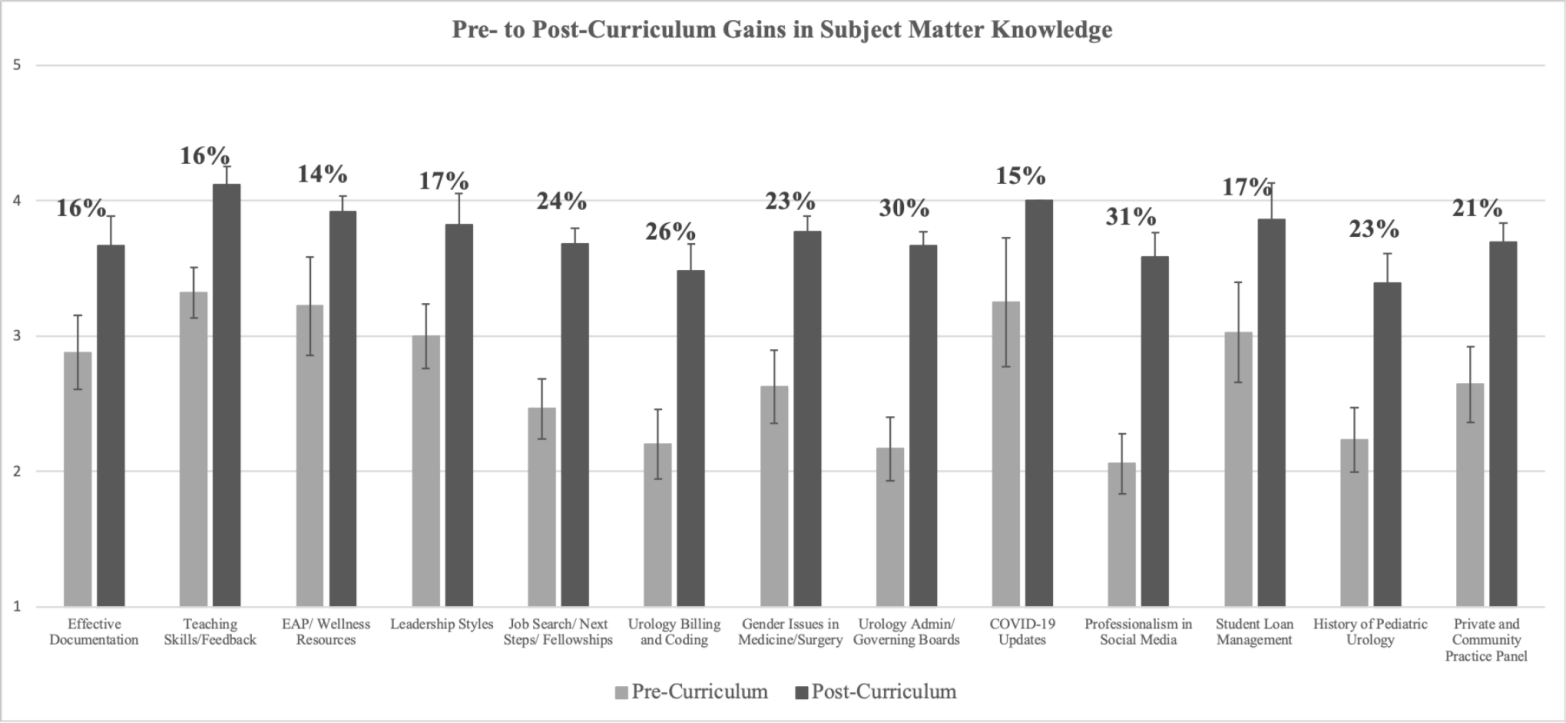
Trainees were asked to self-report pre- and post-curriculum knowledge from learning objectives across each learning session topic via a 5-point Likert scale from 1 (novice) to 5 (expert). Aggregate (mean) pre- and post-curriculum knowledge scores were created for each participant across each learning session. As seen in the figure, participants indicated significant pre- to post-curriculum gains in subject matter knowledge overall (p<0.001) and across each timepoint (all ps<0.001). Reported proportions indicate percent gains from pre- to post-curriculum.

*Gains in Subject Matter Knowledge*: Trainees were asked to rate their knowledge of the subject matter before vs. after participation in each learning session. Broadly, trainees demonstrated significant gains in content knowledge overall (Mean=20%, p<0.001) and across each learning session (**Figure 2**). The greatest gains across learning sessions were observed for junior residents (21%) followed by senior residents (17%).

### Pre- to post-curriculum gains

Trainees indicated small but significant gains on the broad topics that emerged from the pre-curriculum needs assessment. Specifically, they indicated that, following participation in the first-year learning sessions, their level of satisfaction in the amount of NCS training they were currently receiving had increased (p=0.011) and that they were satisfied with this current offering (p=0.047). They continued to report that a high level of NCS training was necessary in both their current role and future careers, and this did not differ from pre-test (M=3.88/5, p=0.351; M=3.50/5, p=1.0). The increase in satisfaction was higher for junior residents (p=0.034) than senior residents.

We also assessed gains in knowledge for specific topics covered in Year 1. Broadly, trainees indicated significant pre- to post-curriculum gains (p=0.004), with the greatest gains seen for Leadership Styles (p=0.033), Urology Administration/Governing Boards (p=0.064), and Teaching/Feedback (p=0.095). Junior and senior residents generally did not differ in their knowledge gains (ps>0.2 except Gender: p=0.034).

### Learner engagement

Although not part of initial study design, based on resident feedback, we decided to investigate degree of learner engagement for each session post-hoc. Though we did not objectively measure degree of trainee interaction for each session we were able to broadly categorize each session as lecture-based or non-lecture based (i.e., interactive in nature) post-hoc. While each session in the curriculum produced statistically significant gains in subject matter, there was consistently higher resident satisfaction for sessions that were more interactive and less didactic or lecture-based, namely Teaching/Feedback, Leadership Styles, Gender and Medicine/Surgery, Preparing for Fellowship, and Private and Community Practice Panel (**Table 2**).

### Curriculum feasibility

Measures of curriculum feasibility included attendance and cost. Attendance ranged from 10 to 14 residents (66.7%-93.3%) for each timepoint curriculum, with 1-2 residents typically out on vacation for that week and 1-2 residents typically tending to patient care emergencies. We estimated the cost of our curriculum at $0 - $1,000 per year. This cost is estimated based on: room rental ($0 at our institution), expert speaker honorarium (we gave $50/speaker), cost of parking ($0 given trainees and speakers already paid for this as members of our institution), and administrative office supplies (<$100/year). Zoom was also provided for free from our institution (virtual sessions).

## Discussion

Here we describe the development, implementation, and evaluation of a novel NCS-based curriculum (e.g., leadership, teaching, professional development, etc.) for urology trainees at our institution as piloted over a two-year timeframe via pre-post test design. In an era of continuous evaluation and feedback-driven implementation of changes to residency curricula, there is a scarcity of literature on this topic pertaining to urology residency programs.

Our novel NCS curriculum was generally well-received by trainees and resulted in statistically significant subjective knowledge gains. While these gains were most notable for junior trainees, there was evidence of improvement in NCS knowledge across all subgroups. The 1-hour sessions were rated highly, which prompted continuation of the curriculum at our institution for a second academic year.

While other leadership curricula have been implemented within surgical residencies (23–26), this study represents the first known reported to the authors designed and tailored specifically to a urology residency, though there are other examples being implemented to address this training gap. For example, the New York Section of the AUA recently ran an 8-part lecture series (available on YouTube) aimed at addressing topics not classically learned in residency, which included a multi-institutional array of guest speakers (27). This series was part of a larger 68-part educational lecture program initiated during the COVID-19 pandemic in an effort to improve resident distance learning, though the cohort was only comprised of 50% residents due to interest from attendings, fellows, and medical students (28). As the need for NCS training in urology is further elucidated, we anticipate the published landscape of this important topic within our specialty will continue to develop and buy-in continue to increase. Notably, there was strong resident buy-in at our institution resulting in a consistent sample group throughout our study.

One reviewer raised an interesting question, that is, whether our graduate medical education office provided any of the education as part of our series. None of our sessions were provided formally by our Office of Graduate Medical Education, however, several of the expert speakers work closely as medical educators with the Office of GME and give similar presentations to other interested departments or in other informal settings. Our group previously worked with the Office of GME at our institution to design, implement, and evaluate a leadership-specific curriculum via similar format. As this manuscript is still under review, we have not cited it here. This unpublished data does show that residents and fellows gained perceived leadership skills following participation in GME-designed content, and this was applicable across specialties. As we have continued to develop this curriculum past this initial implementation, we have continued to work with our institution’s Office of GME to integrate both their educators and available content as relevant (e.g. designing a QI study/fundaments of QI).

We have continued to implement this ‘Hidden Curriculum’ series for our residents since this pilot implementation. As learning has primarily returned to in-person for other conferences, so has this series, with virtual options as needed by speakers, although this is rare. Format and time-wise, the education has remained the same. Sessions continue to be scheduled and coordinated by the Department’s Education Specialist PhD and run by expert speakers in relevant topics, which are either suggested by or reviewed by the Department’s Education Working Group. Based on the feedback in our pilot implementation, some topics are reviewed annually, while some topics will be reviewed only every 2-3 years. As new topics are needed or available, those are added to the rotation and similar metrics are collected for evaluation and continued use.

Given our results, we propose that best practice for curriculum design and implementation – based on our pilot model and some ‘best practices’ developed by our education leadership team – should include a pre-curriculum needs-assessment, protected education time, expert speakers, dedicated support from the residency program coordinator, a pre-post design, and serial learning evaluations to permit continuous feedback and curriculum plasticity. Although these elements are certainly reproducible, the authors recognize that specific topics may vary from program to program and would like to reiterate that a pre-curriculum needs assessment is necessary to optimize the curriculum for individual residency programs.

An additional pre-curriculum tool for educators to assist in building a tailored leadership curriculum used by some studies is the Leadership Practices Inventory (LPI) developed by Posner and Kouzes (29). The results from this tool, which identifies the frequency of 30 leadership behaviors for each participant, may be pooled to identify deficiencies in the curriculum participant cohort and tailor learning sessions to focus on deficient areas.

Although junior and senior residents indicated similar desire for specific topics across the board, unsurprisingly the gaps in knowledge for each session were uniformly more pronounced for juniors. While each session in the curriculum produced statistically significant gains in subject matter, there was consistently higher resident satisfaction for sessions that were more interactive and less didactic or lecture-based. This finding is in agreement with prior published literature suggesting that interactive learning is superior to traditional didactic-based learning in medical education (30). Thus, further development of this curriculum may focus on establishing more interactive sessions.

Though participation was robust and two years of data are reported (i.e., multiple timepoints), knowledge assessment were self-reported and therefore subjective. Common limitations in assessing leadership competencies were thus likely at play in this study including the “honeymoon effect”, “horizon effect”, and “Hollywood effect” (31). Future studies could utilize alternative strategies in leadership assessment including multi-rater evaluations and post-program reflections.

We did not collect demographic data on pathway to medicine (traditional vs non-traditional), presence of medical professionals in the family, and alternate career experience, though these could be interesting variables to examine in future studies to determine whether these factors influence the usefulness/satisfaction of a NCS Curriculum.

Although we did not prospectively include medical students, as a function of our education and rotation schedule, we did have some advanced medical student rotators (n=13, 1-4 per session, single session attendance only) attend these timepoint sessions and asked them to fill out the respective timepoint evaluations, along with our residents. The medical student data is consistent with data collected from residents, indicating that the curriculum was generally felt as useful and well-received. Specifically, reported gains in subject matter knowledge ranged from 22% to 47% (Mean=32%). Medical students similarly reported that the curriculum was beneficial to their education and professional development (Mean=4.2/5), improved their teamwork abilities (Mean=4.1/5), that they would recommend it to a peer (Mean=4.5/5), that it was intellectually challenging (Mean=3.25/5), that it increased their subject matter knowledge (Mean=4.77/5), that it allowed them to practice skills taught in the course (Mean=3.79/5), increased their problem solving skills (Mean=3.88/5), and increased their leadership skills (Mean=3.82/5). Future work should prospectively examine this type of clinical skills education at the medical student level.

Lastly, one could argue that results from this single-institution study may lack generalizability and thus additional future directions include development of a multi-institutional curriculum as well as expanding curriculum topics. The authors do plan on continuing the NCS curriculum at our instution, with plans to cycle through topics every 2-3 years and continue to collect data for quality improvement and curriculum development.

In conclusion, we report the first results (to the authors knowledge) designing, implementing, and assessing an NCS-based curriculum for urology residents. The curriculum was developed to address a critical need within urology resident education and was broadly utilized, well-received, and resulted in measurable subjective gains to NCS and leadership competencies. The ongoing curriculum was affordable, feasible, easily organized, and individualized based on feedback elicited from our trainees. Given its success, the authors recommend similar formal training within urology and other surgery-based residency programs and offer resources for program leadership to institute similar curricula at other institutions.

## Data availability statement

The raw data supporting the conclusions of this article will be made available by the authors, without undue reservation.

## Ethics statement

This study was considered exempt by the Institutional Review Board of the Ohio State University Medical Center. The patients/participants provided their written informed consent to participate in this study.

## Author contributions

All authors have reviewed and approved the final manuscript. All have made meaningful contributions in the study design, statistical analysis and interpretation, and the manuscript drafting and review. All authors contributed to the article and approved the submitted version.
