# Supplementary material for: Implementation and assessment of a novel non-clinical skills curriculum for urology residents
Source: Front Urol. 2023 Jun 13;3:1167966. doi: 10.3389/fruro.2023.1167966 (PMC12327248; doi:10.3389/fruro.2023.1167966)
Supplement: Supplementary file 1 [file DataSheet_1.pdf]

Pre-Hidden Curriculum Assessment

Please fill out the contact information below. Any identifying information will not be associated with your answers on the next page. Thank you for your honesty.

Are you a: Resident Fellow Other: ____________________________

Year: ___________________

Age: ____________________

Gender: Male Female Prefer not to answer

Marital status: Single Married/Living with partner Prefer not to answer

Race/ethnicity:

White

Black or African American

Asian

Native Hawaiian or Other Pacific Islander

American Indian or Alaska Native

Other: _______________________________________

Please answer the following questions about your perceptions and experiences as they relate to your non-clinical skills and training in your current position and program. Answers will be kept anonymous. Thank you for your honesty.

How satisfied/unsatisfied are you with your current non-clinical skills and abilities?

Extremely unsatisfied	Slightly unsatisfied	Neutral	Slightly satisfied	Extremely satisfied

How much non-clinical training is needed to succeed in your current program/position?

None	Slight amount	Moderate amount	Significant amount	Extreme amount

How much non-clinical training is currently provided FORMALLY?

None	Slight amount	Moderate amount	Significant amount	Extreme amount

How much non-clinical training is currently provided INFORMALLY?

None	Slight amount	Moderate amount	Significant amount	Extreme amount

Assuming that it is provided in an optimal method, how much more non-clinical training do you need in preparation for your roles as physician after training?

None	Slight amount	Moderate amount	Significant amount	Extreme amount

How much does your program add to your fund of knowledge regarding non-clinical patient management.

None	Slight amount	Moderate amount	Significant amount	Extreme amount

How much does your program prepare residents/fellows to run a clinic from an administrative perspective.

None	Slight amount	Moderate amount	Significant amount	Extreme amount

→ *Continued on next page*

How much does your program prepare you to handle a large patient volume from an administrative perspective?

None	Slight amount	Moderate amount	Significant amount	Extreme amount

How much does your program prepare you to handle the business aspects of a practice?

None	Slight amount	Moderate amount	Significant amount	Extreme amount

How much does your program help you use other resources in the healthcare system in the management of patients?

None	Slight amount	Moderate amount	Significant amount	Extreme amount

Overall, how satisfied are you currently with your non-clinical training in your program?

None	Slight amount	Moderate amount	Significant amount	Extreme amount

Junior residents should be required to take a non-clinical skills course at least once in their training.

Strongly Disagree	Disagree	Neutral	Agree	Strongly Agree

Senior residents should be required to take a non-clinical skills course at least once in their training.

Strongly Disagree	Disagree	Neutral	Agree	Strongly Agree

Please answer the following questions about your perceptions and experiences as they relate to your leadership skills and training in your current position and program. Answers will be kept anonymous. Thank you for your honesty.

How satisfied/unsatisfied are you with your current leadership abilities?

Extremely unsatisfied	Slightly unsatisfied	Neutral	Slightly satisfied	Extremely satisfied

How much leadership training is needed to succeed in your current program/position?

None	Slight amount	Moderate amount	Significant amount	Extreme amount

→ *Continued on next page*

How much leadership training is currently provided FORMALLY?

None	Slight amount	Moderate amount	Significant amount	Extreme amount

How much leadership training is currently provided INFORMALLY?

None	Slight amount	Moderate amount	Significant amount	Extreme amount

Assuming that it is provided in an optimal method, how much more leadership training do you need in preparation for your roles as physician after training?

None	Slight amount	Moderate amount	Significant amount	Extreme amount

Overall, how satisfied are you currently with your leadership training in your program?

None	Slight amount	Moderate amount	Significant amount	Extreme amount

I have a good understanding of different leadership styles.

Strongly Disagree	Disagree	Neutral	Agree	Strongly Agree

My knowledge of leadership styles will inform my interactions with my clinical teammates.

Strongly Disagree	Disagree	Neutral	Agree	Strongly Agree

Junior residents should be required to take a Leadership Training Program at least once in their training.

Strongly Disagree	Disagree	Neutral	Agree	Strongly Agree

Senior residents should be required to take a Leadership Training Program at least once in their training.

Strongly Disagree	Disagree	Neutral	Agree	Strongly Agree

I have a need for training in leadership competencies.

Strongly Disagree	Disagree	Neutral	Agree	Strongly Agree

→ *Continued on next page*

For whom would a formal non-clinical skills curriculum be most relevant?
(*Check all that apply*):

_____ Medical Students

_____ Interns (PGY1s)

_____ Junior Residents (~PGY2/3)

_____ Senior Residents (~PGY4 and higher)

______ Chief Residents

_____ Fellows

Please rate your knowledge of the following topics on a scale of 1-5:

1=Novice 2=Low proficiency 3=Average 4=High proficiency 5=Expert

_____ Effective documentation

_____ Advanced directives and delivering bad news

_____ Teaching skills, eliciting feedback

_____ Well-being/wellness/maintaining passion

_____ Understanding MOC, CME, and professional responsibilities

_____ Leadership Styles

_____ Common patient complaints and strategies to prevent them

_____ How to conduct a job search; finding fellowships/next steps

_____ Cultural competency

_____ Bioethics (autonomy, justice, beneficence, maleficence)

_____ Informed consent

_____ Urology billing and coding

_____ Gender issues in medicine/surgery

_____ Urology administration (history and explanations of differing governing boards)

→ *Continued on next page*

Are there any topics not listed above (and not currently addressed in other curricula) that you would like to see covered during Hidden Curriculum?

You're all done! Thank you for your time!
