# Supplementary material for: Implementation and assessment of a novel non-clinical skills curriculum for urology residents
Source: Front Urol. 2023 Jun 13;3:1167966. doi: 10.3389/fruro.2023.1167966 (PMC12327248; doi:10.3389/fruro.2023.1167966)
Supplement: Supplementary file 2 [file DataSheet_2.pdf]

Department of Urology: Hidden Curriculum **February 20, 2020**

Topic: Compliance, Coding, Evaluation and Management Services

Name: ______________________________

I am a: (*please circle*) Resident Fellow Other: ___________________________

PGY: __________

Please rate your <u>knowledge</u> of the following topics **before** vs. **after** today's curriculum on a scale of 1-5:<br>**1= novice 2=low 3=average 4=above average 5=expert**	**Before today:**	**After today:**
Guidelines and documentation for teaching physician billing		
What the global surgical package refers to		
Global surgical package guidelines		
Modifier usage		
Modifier payment/billing based on usage		
Definition of a group practice		
Consultation guidelines		
The "Incident to" provision of Medicare		
Understanding of "medical necessity"		
Understanding of 3 components of E/M: History, Exam, Medical Decision Making		
Outpatient billing guidelines		

<u>**Please rate the following for TODAY'S workshop:**</u>

(1) Today's curriculum helped me better understand teaching physician compliance.

Disagree Somewhat Disagree Neutral Somewhat Agree Agree

(2) Today's curriculum helped me better understand global surgery (as it relates to billing/coding).

Disagree Somewhat Disagree Neutral Somewhat Agree Agree

(3) Today's curriculum helped me better understand modifiers.

Disagree Somewhat Disagree Neutral Somewhat Agree Agree

→ *Continue on back*

(4) Today's curriculum helped me better understand Evaluation and Management Guidelines.

Disagree	Somewhat Disagree	Neutral	Somewhat Agree	Agree

(5) Today's curriculum helped me better understand different leadership styles.

Disagree	Somewhat Disagree	Neutral	Somewhat Agree	Agree

(6) Today's curriculum was beneficial to my ability to work with a team.

Disagree	Somewhat Disagree	Neutral	Somewhat Agree	Agree

(7) I would recommend today's curriculum to my peers.

Disagree	Somewhat Disagree	Neutral	Somewhat Agree	Agree

(8) Today's curriculum was intellectually challenging.

Disagree	Somewhat Disagree	Neutral	Somewhat Agree	Agree

(9) Today's curriculum increased my knowledge of the subject matter.

Disagree	Somewhat Disagree	Neutral	Somewhat Agree	Agree

(10) Today's curriculum provided me with the opportunity to practice the skills taught in the course.

Disagree	Somewhat Disagree	Neutral	Somewhat Agree	Agree

(11) Today's curriculum improved my problem-solving skills.

Disagree	Somewhat Disagree	Neutral	Somewhat Agree	Agree

(12) Today's curriculum improved my leadership skills.

Disagree	Somewhat Disagree	Neutral	Somewhat Agree	Agree

What did you learn today? ____

____

____
